# Cytotoxic activity of settled dust in primary schools: a multi-matrix, multi-season screening approach for children’s environmental health

**DOI:** 10.3389/fpubh.2026.1946465

**Published:** 2026-10-07

**Authors:** Renata Cervantes, Pedro Pena, Elisabete Carolino, Ewelina Soszczyńska, Magdalena Twarużek, Susana Viegas, Carla Viegas

**Affiliations:** 1 NOVA National School of Public Health, Public Health Research Center, Comprehensive Health Research Centre, CHRC, REAL, CCAL, NOVA University Lisbon, Lisbon, Portugal; 2 H&TRC—Health & Technology Research Center, ESTeSL—Escola Superior de Saúde, Instituto Politécnico de Lisboa, Lisbon, Portugal; 3 Department of Physiology and Toxicology, Faculty of Biological Sciences, Kazimierz Wielki University, Bydgoszcz, Poland

**Keywords:** children's environmental health, cytotoxicity, fungal contamination, indoor air quality (IAQ), passive sampling, school environment, settled dust

## Abstract

Children spend up to nine hours daily in school environments, yet the potential health impact of accumulated indoor dust remains largely uncharacterised. This study provides the first multi-matrix, multi-season assessment of passive dust samples from Portuguese primary schools, using the MTT assay to evaluate the biological activity of complex indoor pollutant mixtures. 207 passive samples were collected across 11 primary schools in the Lisbon Metropolitan Area, using four sampling matrices, settled dust filters, electrostatic dust cloths (EDC), electrostatic dust cloths on T-shirts (EDCT), and floor mops, across six indoor locations, warm and cold seasons, and in urban and rural contexts. Cytotoxic activity was assessed in A549 (human alveolar epithelial) and SK (swine kidney) cells, potential azole resistance was detected by growth on azole-supplemented culture media. Cytotoxic activity was detected in 64.7% (A549) and 24.6% (SK) of samples. Sampling matrix was the strongest predictor of cytotoxic variability for both cell lines (A549: R^2^ = 0.199; SK: R^2^ = 0.081; *p* < 0.001), with floor mops exhibiting the highest cytotoxic rates (A549: 94.4%; SK: 83.3%). Season was a significant secondary modulator exclusively for A549 cells, with cold-season positivity of 82.8% versus 50.0% (*p* < 0.001). Geographic context, school identity, and indoor location showed no significant effects, suggesting cytotoxic variability reflects building-level operational factors, cleaning practices and ventilation, rather than environmental setting. Spearman analysis revealed cell-line and compound-specific associations between fungal burden, potential azole resistance detected by growth on azole-supplemented culture media, and cytotoxic responses, with reduced susceptibility to itraconazole, voriconazole, and posaconazole detected in school dust isolates. These findings demonstrate that microbial contamination in school settled dust may result in potential health effects involving clinically relevant antifungal resistance species. Floor mop sampling is identified as a high-sensitivity sentinel approach for the combined biological and chemical toxic burden accumulated at floor level. Passive multi-matrix cytotoxicity assessment should be integrated into school indoor air quality surveillance to protect this vulnerable population.

## 1 Introduction

The World Health Organization (WHO) recognized the fundamental right to healthy indoor air nearly two decades ago (1), prompting the inclusion of indoor air quality (IAQ) as a regional priority in the Children’s Environmental Health Action Plan for Europe (CEHAPE) (2). Children, as a vulnerable population, spend between 6 to 9 h daily in school buildings (3), making the school environment a critical setting for environmental health assessment. Over the years, a growing body of research has highlighted the adverse health effects of poor IAQ in schools, linking exposure to indoor pollutants with respiratory symptoms, allergic reactions, and other chronic conditions (4–6). However, significant heterogeneity in IAQ regulations across Europe, including gaps in policies targeting microbial and chemical contaminants, has hindered the development of harmonized mitigation strategies (7). In response to these challenges, a study was conducted in 11 primary schools in Portugal in the scope of InChildHealth project,^1^ covering 28 classrooms, to assess the indoor environmental quality using passive sampling techniques. Four types of samples: settled dust vacuum filters, electrostatic dust cloths (EDC), electrostatic dust cloths in T-shirts (EDCT), and floor mops, were collected to provide a comprehensive overview of accumulated pollutants in indoor environments. These sampling matrices offer long-term integrative data, particularly relevant in educational settings that often lack active environmental monitoring systems (8, 9).

Building on cytotoxicity methods previously validated in occupational settings (10, 11), this study applies the MTT assay for the first time to a multi-matrix, multi-season assessment of passive samples from Portuguese primary schools, an environment where children represent the primary exposed group. Cell viability was assessed using two cell lines: human alveolar epithelial cells (A549), selected as the most physiologically relevant model for pulmonary exposure via inhalation; and swine kidney cells (SK), included as a comparative tissue model to characterise differential cytotoxic responses across cell types (10, 12). The use of two distinct cell lines enables a more comprehensive characterisation of the potential health effects posed by the complex pollutant mixtures present in indoor dust, supporting its application as a screening tool for potential health effects assessment. Beyond the direct cytotoxic effects of fungal contaminants, growing evidence points to antifungal resistance as an additional and underappreciated concern in non-clinical indoor environments. Azole resistance in *Aspergillus fumigatus* has become a global public health threat, with resistance detected in 15–20% of isolates in parts of Europe and exceeding 80% in environmental samples in Asia (13). Environmental reservoirs, including dust accumulated in indoor spaces, have been identified as potential sources of azole-resistant fungal strains, with built environments, notably hospitals and gardens, emerging as the most frequent hotspots, followed by agricultural soils (14–16). Recent multi-agency assessment by EU health and environment agencies confirmed that environmental hotspots for azole resistance selection include agricultural settings with stockpiled agricultural waste and their use as soil amendments, with approximately 120,000 tonnes of azoles used annually in the EU/EEA outside human medicine (17). The detection of reduced susceptibility to itraconazole, voriconazole, and posaconazole in environmental isolates is particularly concerning in school settings, where immunocompromised children or those with underlying respiratory conditions can be present (18). Despite the established role of these environmental reservoirs in the dissemination of resistant strains, school buildings, particularly those in rural or peri-urban areas near agricultural land, remain largely uninvestigated as potential hotspots for resistant fungal contamination. Therefore, characterising not only the cytotoxic potential but also the antifungal resistance profiles of fungal contaminants recovered from schools represents a critical and underexplored dimension of children’s environmental health surveillance. Moreover, climate change is increasingly recognized as a contributing factor to indoor environmental degradation (19). Rising temperatures, changes in humidity, and more frequent extreme weather events (e.g., floods) may affect building conditions and promote the emissions and accumulation of indoor contaminants (20, 21). These climate-driven shifts may amplify the toxic potential of indoor environments, especially in old school infrastructure (22, 23). From a broader perspective, climate change is profoundly influencing the prevalence, distribution, and toxicity of environmental fungi (24, 25). Increased atmospheric CO2, higher temperatures, and altered precipitation patterns can enhance fungal growth and sporulation in both outdoor and indoor environments (26). These conditions may lead to an increased fungal contamination in school settings and potentially shift the mycobiota towards more xerophilic or thermotolerant species (27). Furthermore, climate stressors can modify fungal metabolism, potentially increasing the production and release of mycotoxins and other cytotoxic compounds (18, 28). This heightened fungal burden and altered toxicity pose potential health concerns, as exposure to fungal contaminants may result in a range of adverse effects, including respiratory inflammation, allergic sensitization, and cytotoxic damage of epithelial cells, which can be particularly detrimental to children’s developing respiratory systems (29, 30). This study aims to: (i) evaluate the cytotoxic potential of environmental samples collected by passive methods in Portuguese primary schools; (ii) assess whether cytotoxic activity differs between urban and rural school environments; (iii) examine the seasonal variability in fungal burden and cytotoxic potential; (iv) identify which passive sampling matrix most effectively discriminates differences in indoor microbial contamination; and (v) determine whether fungal strains with reduced antifungal susceptibility are detectable in the school environment, an aspect with direct implications for the clinical management of vulnerable school-age populations.

## 2 Materials and methods

This study is part of the European InChildHealth project (EU Horizon 2021, grant 101056883), a multidisciplinary initiative addressing determinants of indoor air quality (IAQ) affecting school-aged children (6–12 years). Between June 2023 and September 2024, environmental assessments were conducted in eleven elementary schools across urban and rural areas of the Lisbon Metropolitan Area, selected for geographic diversity and enrolment of children aged 6–12 years (Figure 1).

**Figure 1:**
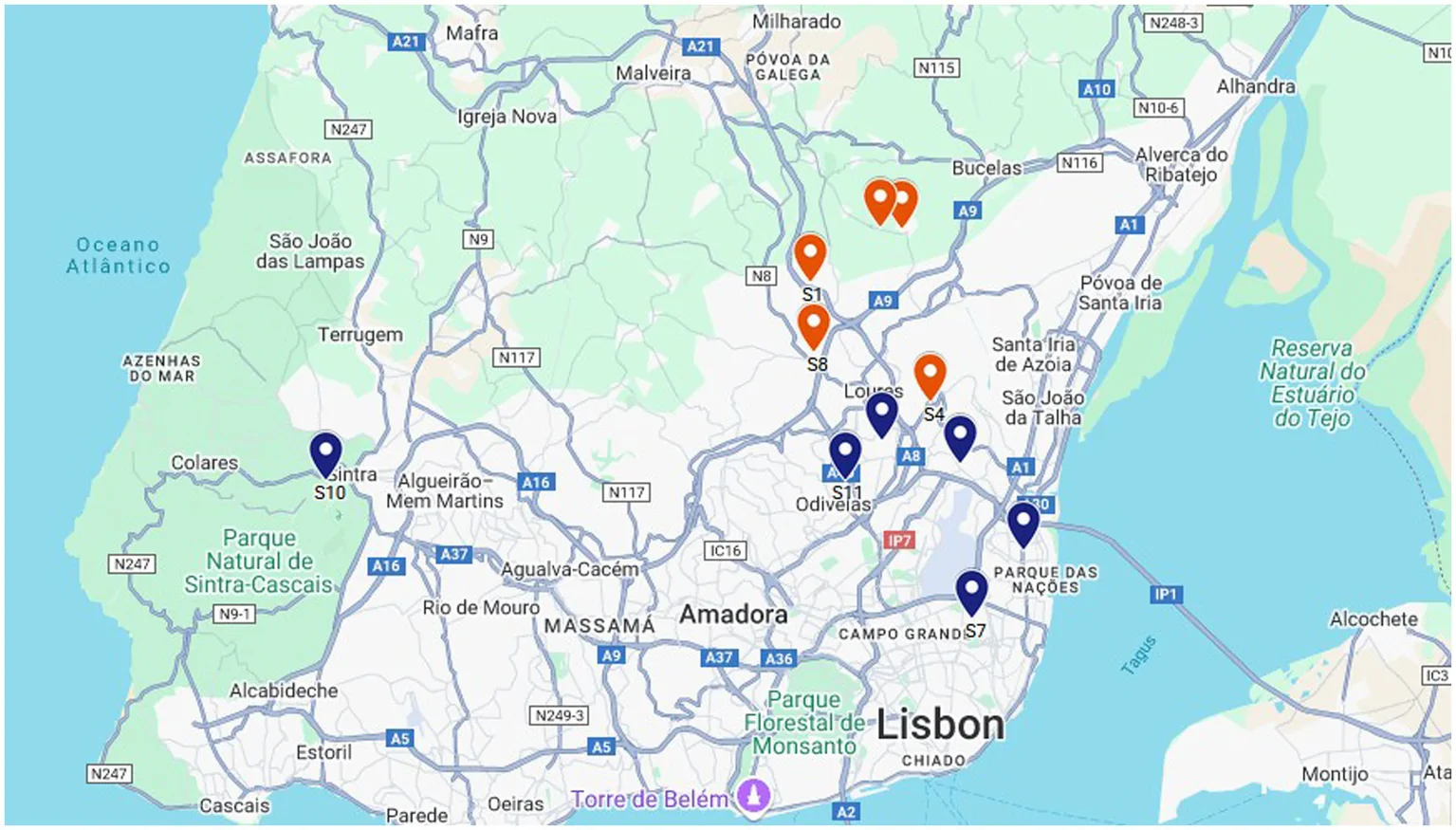
Geographical location of the sampled schools. The locations were chosen to cover different areas, including urban contexts (marked in blue) and rural or semi-rural contexts (marked in red).

### 2.1 Contextual information

School contextual and operational information (cleaning procedures, classroom occupancy, staff numbers, ventilation type, presence of animals or plants, and time children spent indoors) and full environmental sampling protocols have been detailed previously (31), and are summarised in Supplementary material (Section S1, Supplementary Table S1). In brief, sampling was conducted across six locations per school (classrooms, canteens, libraries, bathrooms, gymnasiums, and playgrounds) during regular school hours, using four passive dust-accumulating matrices: vacuum settled-dust filters, electrostatic dust cloths (EDC), EDC worn on children’s T-shirts (EDCT), and mops collected at the end of the routine wet-cleaning cycle, in the used state and before reprocessing. Mops were sampled as sentinel collectors integrating floor-level contamination across high-traffic surfaces; because collection follows the application of cleaning products, mop extracts represent the combined biological (fungal biomass, spores and secondary metabolites) and chemical (detergent and disinfectant residue) burden present at floor level, to which both children and cleaning staff are exposed. EDCs were deployed for 30 consecutive days. All samples were stored in sterile bags at 0–4 °C until analysis, following validated protocols adapted from Viegas et al. (32). The overall study workflow is summarised in Supplementary Figure S1.

### 2.2 Sample extraction and microbiological analysis

Sample extraction was performed as previously described (32). Briefly, samples were washed with a 0.1% Tween 80 saline solution (0.9% NaCl) under agitation (250 rpm for 30 min), using volumes adjusted according to sample type (settled dust filters, mops, electrostatic dust collectors, composite settled dust). The resulting extracts were used for fungal quantification by culture-based methods (33), morphological identification, and potential azole resistance detection by growth on azole-supplemented media (34), as detailed in previous work (32, 35). Azole susceptibility was assessed here by phenotypic screening only; MIC determination and molecular characterisation of resistance determinants (CYP51A sequencing and promoter tandem-repeat detection) fall outside the scope of the present analysis and will be reported separately. The resistance profiles used in the correlation analyses below are therefore phenotypic and presumptive. The microbiological data (CFU·m^−2^, CFU·m^−2^·day^−1^, fungal species, azole susceptibility profile) were used in the correlation analyses with cytotoxicity.

A comprehensive screening was conducted for all samples to assess the presence of mycotoxins. Mycotoxins detection was conducted through high-performance liquid chromatography (HPLC) using the Nexera system (Shimadzu, Tokyo, Japan), coupled with a triple quadrupole-linear ion trap mass spectrometer (5,500 Qtrap; Sciex, Foster City, CA, United States). The specific mycotoxins analysed, along with their corresponding limits of detection (LOD) and limits of quantification (LOQ), are reported in Cervantes et al. (31).

### 2.3 Cytotoxicity analysis

To evaluate the cytotoxicity of microbial contaminants recovered through passive sampling, swine kidney (SK) and A549 (human alveolar epithelial) cell lines were used. Cells were cultured at 37 °C in Eagle’s Minimum Essential Medium (MEM) supplemented with 10,000 units of penicillin, 10 mg/mL streptomycin, and fetal calf serum (FCS, Sigma-Aldrich, United States). For subculturing, cells were detached from the culture flasks by trypsinization using 0.25% (w/v) trypsin with 0.53 mM EDTA. The resulting cell suspensions were resuspended in fresh culture medium and adjusted to concentrations of 2.0 × 10^5^ cells/mL for SK and 3.0 × 10^5^ cells/mL for A549, as determined using a Scepter™ 2.0 Cell Counter (Merck). Aliquots of 100 μL of each cell suspension were dispensed into individual wells of 96-well plates and exposed to serial 1:2 dilutions of the sample extracts. The plates were then incubated for approximately 48 h at 37 °C in a humidified atmosphere containing 5% CO₂. Following incubation, the culture medium was removed, and the MTT (3-(4,5-dimethylthiazol-2-yl)-2,5-diphenyltetrazolium bromide) assay was performed as previously described (36) to determine cell proliferation and viability. The optical density (OD) of each well was measured spectrophotometrically using an ELISA reader (ELISA LEDETECT 96, biomed Dr. Wieser GmbH) equipped with MikroWin 2013SC software, at a wavelength of 510 nm. The threshold toxicity level (IC₅₀) was defined as the lowest concentration resulting in a reduction of cell metabolic activity to less than 50%. Samples not reaching the IC₅₀ threshold at any tested dilution were classified as non-cytotoxic and excluded from quantitative comparisons.

### 2.4 Statistical analysis

The R-Studio software version 4.3.3 was used to analyse the data. The results were considered significant at the 5% significance level. To test the univariate normality of the data, the Shapiro–Wilk test was used (R command: shapiro.test(var)). To test multivariate normality, the Henze-Zirkler (HZ) test (R command: mvn(data, mvnTest = “hz”)) and Mardia test (R command: mvn(data, mvnTest = “mardia”)) were used. To evaluate the effect of the combined factors Context, Matrices, School, Season, and Location on cytotoxicity in A549 and SK cells at different dilutions (1 to 5), PERMANOVA (Permutational Analysis of Variance) based on Euclidean distances (R command: adonis2()) was used, since multivariate normality was not verified. To test the internal variability of the groups, the PERMDISP test (Permutational Analysis of Multivariate Dispersals—command betadisper (dist, group)) was used. In cases where statistically significant differences were detected, to identify the most influential dilution in both A549 and SK cells, SIMPER analysis (Similarity Percentage analysis) was used. The NMDS (Non-metric Multidimensional Scaling) order was used to visualize multivariate cytotoxicity profiles. The NMDS axes represent abstract gradients that summarize dissimilarities between samples; distances between points reflect relative differences in cytotoxicity profiles. To study the relationship between fungal growth on azole-supplemented media with cytotoxicity in A549 and SK cells at five different dilutions, Spearman’s correlation coefficient (R command: rcorr(as.matrix(data), type = “spearman”)) was used, since the assumption of normality was not met. Multivariate PERMANOVA analyses were performed to evaluate the combined and individual effects of context (urban vs. rural), sampling matrix, school, season, and location on cytotoxicity profiles in A549 and SK cells across five serial dilutions (1 to 5). Multivariate normality was not confirmed by the Henze-Zirkler and Mardia tests (*p* < 0.05), supporting the use of permutation-based methods throughout.

## 3 Results

### 3.1 Cytotoxicity

A total of 207 environmental samples were screened for cytotoxic activity using the MTT assay in two cell lines: human alveolar epithelial cells (A549) and swine kidney cells (SK). Cytotoxic activity was detected in 64.7% of samples using A549 cells and in 24.6% using SK cells at the lowest tested dilution (1:2); 29.5% of samples were non-cytotoxic across all dilutions (Table 1). A549 cells showed a steeper dose–response gradient with increasing dilution compared to SK cells, which maintained more stable positivity rates across dilutions, suggesting greater inter-sample variability in cytotoxic response for the latter. Among positive samples, A549 cell viability ranged from 2.5 to 49.9% (median 38.5%; IQR 32.1–44.1%), while SK cell viability showed a wider spread (2.1–50.1%; median 42.1%; IQR 15.5–46.1%). Cytotoxic positivity varied by sampling matrix, school location, season, and geographic context, as detailed in Table 1 and Figure 2. Notably, mop samples and bathroom locations showed the highest positivity rates for both cell lines, while A549 cytotoxicity was markedly higher in the cold season. Statistical comparisons between groups are presented in section 3.2.

**Table 1:** Cytotoxic positivity rates of A549 and SK cells at dilution 1 (1:2) by sample category (*N* = 207).

Category	*n*	A549 positive — *n* (%)	SK positive — *n* (%)
Overall	**207**	134 (64.7%)	51 (24.6%)
Sampling matrix			
Settled dust filter	93	63 (67.7%)	15 (16.1%)
Electrostatic dust cloth (EDC)	60	27 (45.0%)	17 (28.3%)
Electrostatic dust cloth on T-shirt (EDCT)	36	27 (75.0%)	4 (11.1%)
Floor mop (MOP)	18	**17 (94.4%)**	**15 (83.3%)**
Context			
Urban	113	72 (63.7%)	26 (23.0%)
Rural	94	62 (66.0%)	25 (26.6%)
Season			
Warm	114	57 (50.0%)	35 (30.7%)
Cold	93	**77 (82.8%)**	16 (17.2%)
School location			
Bathroom	13	**11 (84.6%)**	**11 (84.6%)**
Canteen	27	19 (70.4%)	9 (33.3%)
Classroom	133	83 (62.4%)	24 (18.0%)
Gymnasium	11	7 (63.6%)	2 (18.2%)
Library	23	14 (60.9%)	5 (21.7%)

Bolded cells indicate positivity rates ≥ 80%. Positivity defined as cell viability < 50% (IC_50_) at dilution 1 (1:2). Statistical comparisons between groups are presented in section 3.2.

**Figure 2:**
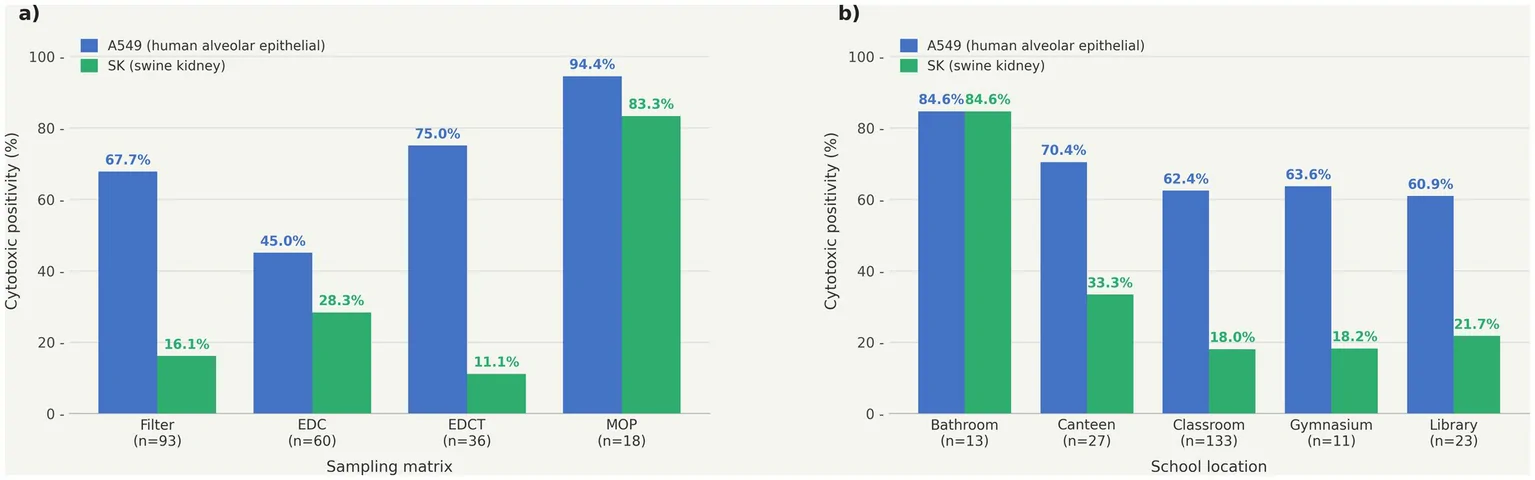
Cytotoxic positivity rates (%) of A549 (human alveolar epithelial) and SK (swine kidney) cells at the lowest tested dilution (1:2). **(a)** By school location; **(b)** By sampling matrix. Positivity defined as cell viability < 50% (IC₅₀). Sample sizes indicated below each category label.

The elevated cytotoxicity observed in bathroom locations and mop samples may reflect exposure to cleaning product residues in addition to microbial contamination, as these environments typically receive more intensive chemical cleaning protocols.

### 3.2 Correlation and comparison analyses

#### 3.2.1 A549 cells

The global PERMANOVA model was significant for A549 cells (R^2^ = 0.365, *F* = 5.652, *p* < 0.001, Supplementary Table S2). Marginal analysis (Supplementary Table S3) revealed that only sampling matrix (R^2^ = 0.199, *p* < 0.001) and season (R^2^ = 0.071, *p* < 0.001) independently explained cytotoxic variation. Context, school, and location showed no significant effects (all *p* > 0.05). Matrix effects were complex, involving both centroid shifts and dispersion differences (PERMDISP *p* < 0.05). NMDS ordination (Figure 3a) confirmed that MOP samples occupied a distinct, widely dispersed region, while EDC and EDCT samples clustered tightly together. SIMPER analysis identified the two lowest dilutions (A549_1, A549_2) as primary contributors to between-matrix dissimilarity (60–92% of cumulative dissimilarity, Supplementary Table S4). For season, these same two dilutions jointly explained ~71% of warm-cold dissimilarity, with NMDS ordination confirming comparable internal variability between seasons (Figure 4a).

**Figure 3:**
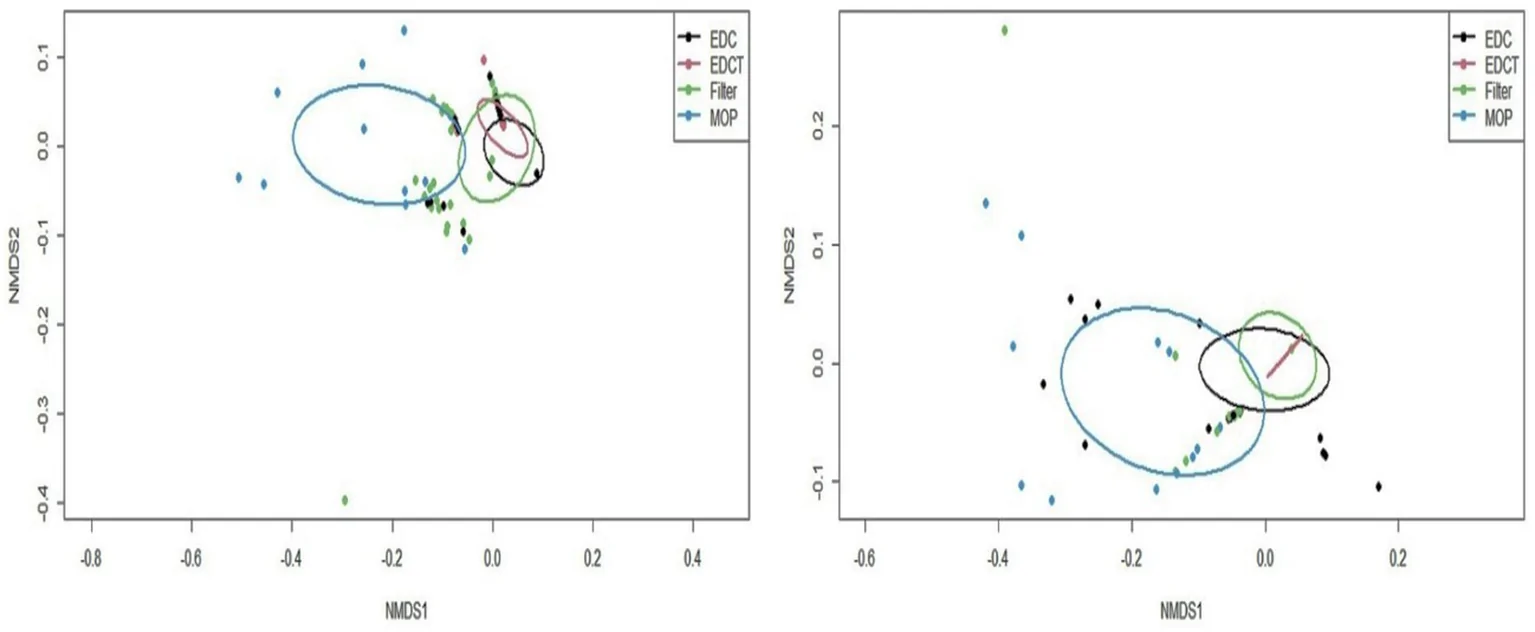
NMDS ordination of cytotoxicity profiles across five dilutions, by sampling matrix. **(a)** A549 cells; **(b)** SK cells. Ellipses represent one standard deviation around group centroids, illustrating within-matrix variability. Filter: settled dust filters; EDC: electrostatic dust cloths; EDCT: electrostatic dust cloths on children’s T-shirts; MOP: floor mops.

**Figure 4:**
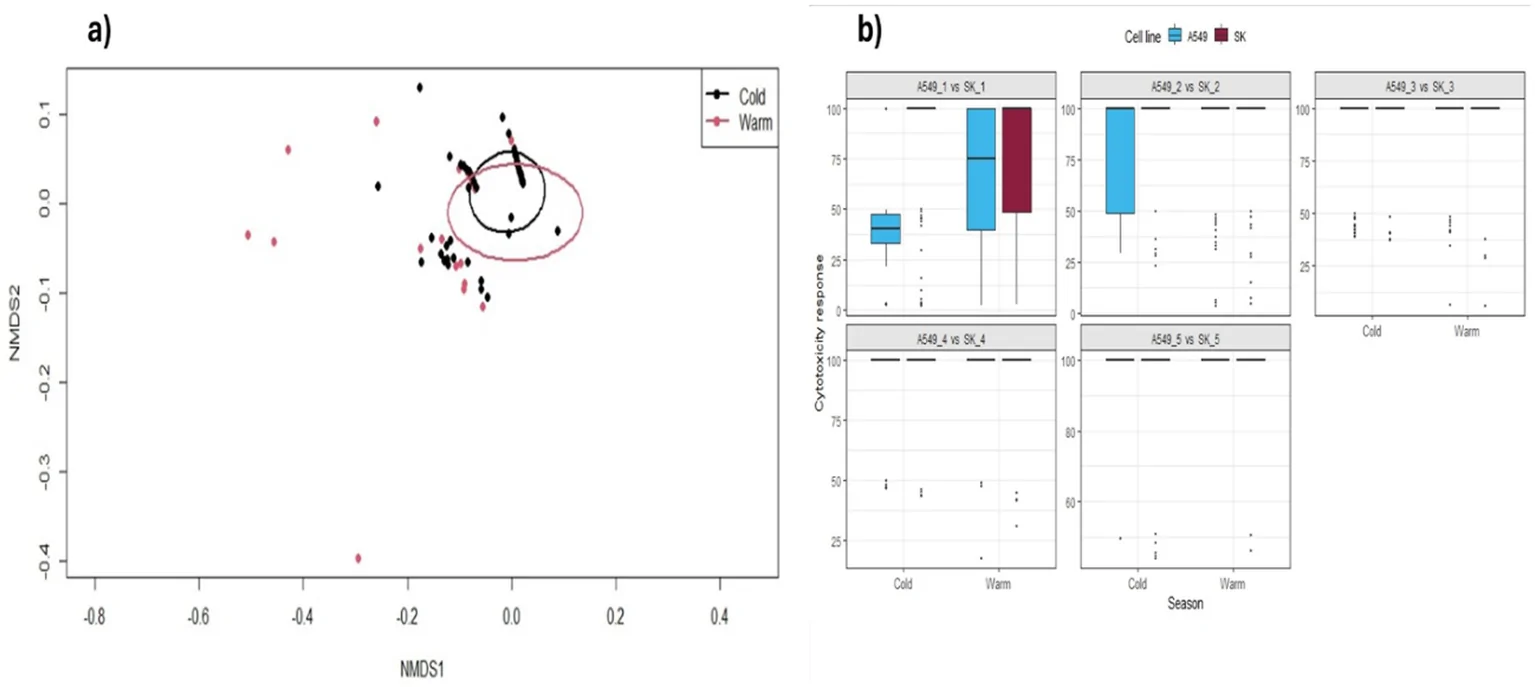
Seasonal cytotoxicity profiles. **(a)** NMDS ordination of A549 cytotoxicity profiles (dilutions 1–5) by season. Ellipses represent one standard deviation around group centroids. **(b)** Comparison of cytotoxicity at different dilutions between A549 and SK cells, by season.

#### 3.2.2 SK cells

*The global* model was also significant for SK cells, albeit with lower explanatory power (R^2^ = 0.294, *F* = 4.091, *p* < 0.001) (Supplementary Table S5). Marginal analysis (Supplementary Table S6) showed that, in contrast to A549 cells, only sampling matrix emerged as a significant predictor (R^2^ = 0.081, *p* < 0.001). Season, context, school, and location were not significant (all *p* > 0.05). PERMDISP confirmed heterogeneity of matrices (*p* < 0.001), again driven by MOP samples. NMDS ordination (Figure 3b) confirmed this pattern, showing MOP as the most dispersed group. SIMPER analysis (Supplementary Table S7) showed that dilution 1 (SK_1) alone accounted for 52–54% of dissimilarity in MOP vs. filter and MOP vs. EDCT comparisons.

#### 3.2.3 Comparative profiles and between-cell-line differences

Sampling matrix was the most discriminating factor for both cell lines (Figure 5). MOP samples consistently showed the lowest median viability (greatest cytotoxicity) and widest interquartile ranges, while EDC/EDCT samples displayed overlapping, homogeneous distributions. A key cell-line divergence was observed for season (Figure 4b): A549 cells exhibited markedly lower viability during the cold season, consistent with the significant PERMANOVA effect (Supplementary Table S3), whereas SK cells showed no comparable seasonal trend (Supplementary Table S6), indicating differential sensitivity between alveolar epithelial (A549) and swine kidney (SK) cells (Supplementary Figure S2). Collectively, the sampling matrix is the primary determinant of indoor cytotoxic activity, irrespective of cell line. Season constitutes a secondary, cell-line-specific modulator relevant for respiratory health surveillance. The absence of urban–rural differences (Supplementary Figure S3) suggests that indoor fungal contamination in schools is driven more by building-level factors and cleaning practices than by the surrounding environmental context. No consistent site-dependent pattern was shown, with elevated values appearing sporadically across locations (Supplementary Figure S4). Despite the variability observed between schools, no consistent pattern of higher or lower cytotoxicity associated with specific schools is identified. The differences appear to reflect local variability, not a systematic effect (Supplementary Figure S5).

**Figure 5:**
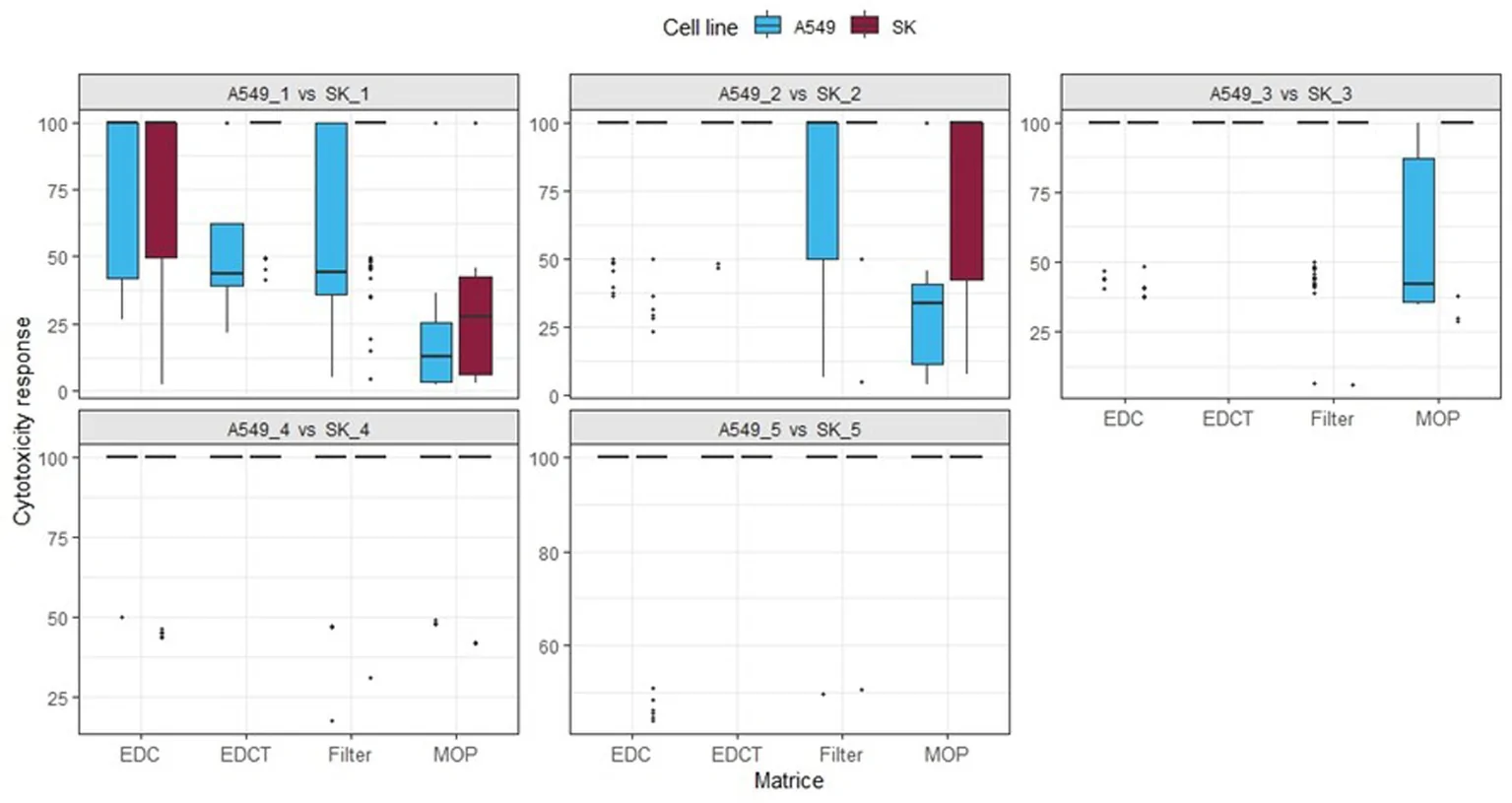
Boxplot comparison of cytotoxicity at different dilutions between A549 and SK cells, by sampling matrix. Filter: settled dust filters; EDC: electrostatic dust cloths; EDCT: electrostatic dust cloths on children’s T-shirts; MOP: floor mops. Lower values indicate greater cytotoxic activity.

#### 3.2.4 Spearman correlation with fungal growth and azole resistance

Spearman correlation analysis (Figure 6) revealed contrasting association patterns. Fungal growth showed consistent positive correlations with SK cytotoxicity (most prominent at SK_5, ρ up to 0.27, *p* < 0.01) but negative correlations with A549 cytotoxicity (A549_1, ρ as low as −0.22, *p* < 0.05). Potential azole resistance displayed compound-specific profiles: voriconazole (VCZ) resistance correlated negatively with A549 cytotoxicity across multiple dilutions, while itraconazole (ITZ) and posaconazole (PSZ) resistance were predominantly associated with SK responses.

**Figure 6:**
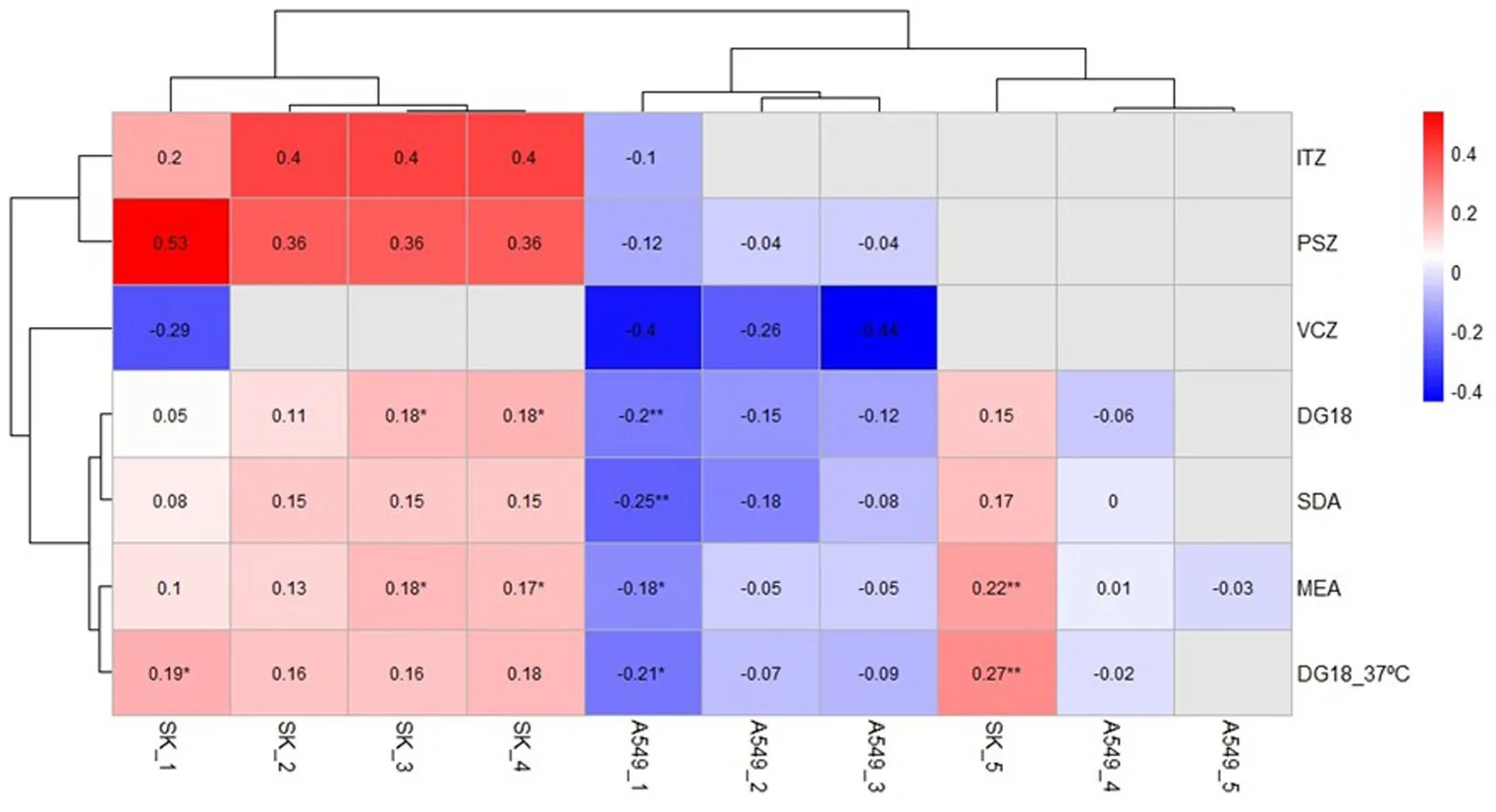
Spearman correlation matrix and dendrogram illustrating associations between fungal growth variables, potential azole resistance (VCZ: voriconazole; ITZ: itraconazole; PSZ: posaconazole), and cytotoxicity at five dilutions in A549 and SK cells. Positive correlations shown in warm tones; negative correlations in cool tones. * *p* < 0.05; ** *p* < 0.01.

## 4 Discussion

### 4.1 Cytotoxic potential of passive environmental samples in Portuguese primary schools

Cytotoxic activity was detected in 64.7% of samples using A549 cells and 24.6% using SK cells, rates broadly consistent with, and in several instances exceeding, those reported in comparable occupational settings. Studies in Portuguese carpentries and DIY stores using the same dual cell-line MTT protocol reported A549 positivity ranging from 50.6 to 84.9% depending on season and matrix (37, 38) while assessments in Norwegian waste sorting facilities found cytotoxicity in a minority of filter samples but high positivity in settled dust (39). The higher A549 sensitivity observed throughout aligns with the physiological relevance of this alveolar epithelial model for respiratory inhalation exposure (12), and with findings by Lu et al., who demonstrated strong species- and isolate-level variance in cytotoxic potential among *Penicillium* and *Aspergillus* species isolated from nursing home cleaning workers, noting no obligatory relationship between inflammatory and cytotoxic potentials (40). The greater interquartile range of SK cell viability (IQR 15.5–46.1%) compared to A549 (IQR 32.1–44.1%) reflects higher heterogeneity in renal cell responses, reinforcing the value of including multiple cellular models in environmental cytotoxicity assessments to capture the full spectrum of biological activity in complex indoor pollutant mixtures.

### 4.2 Urban–rural differences in cytotoxic activity

Neither geographic context nor school identity reached statistical significance for either cell line, indicating that building-level factors, ventilation, cleaning practices, and occupancy, rather than surrounding environmental context, drive cytotoxic exposure in this setting. This finding is consistent with a systematic review of forty-seven studies on school fungal contamination, which found that intra-regional within-setting variability dominates local IAQ profiles over geographic gradients (4), and implies that monitoring and mitigation efforts should prioritize operational and infrastructural interventions over geographic stratification.

### 4.3 Seasonal variability in fungal burden and cytotoxic potential

Season was a significant predictor of A549 cytotoxicity (R^2^ = 0.071, *p* < 0.001), with cold-season positivity of 82.8% vs. 50.0% in the warm season but was non-significant for SK cells (*p* = 0.517). This divergence likely reflects the A549 model’s heightened sensitivity to seasonal shifts in indoor fungal composition: reduced natural ventilation during colder months in Portuguese schools promotes bioaerosol accumulation, and cold-season indoor fungal communities tend toward more xerophilic and toxigenic species with enhanced secondary metabolite output (10, 41). Similar seasonal amplification of cytotoxicity in A549 cells was documented in carpentries, where filters from vacuumed dust drove cold-season cytotoxic differences (38). The absence of a seasonal signal in SK cells supports the use of A549 as the preferential model for respiratory health assessment in indoor environments occupied by children.

### 4.4 Sampling matrix as the most discriminating approach for indoor microbial contamination

Sampling matrix was the strongest independent predictor of cytotoxic variability for both cell lines (A549: R^2^ = 0.199, *p* < 0.001; SK: R^2^ = 0.081, *p* < 0.001), with floor mops consistently exhibiting the highest positivity rates (A549: 94.4%; SK: 83.3%). This result corroborates the growing evidence that matrix type profoundly shapes the detected biological activity of indoor dust (9, 42), and is mechanistically explicable: unlike electrostatic passive collectors, mops used in wet school floor cleaning accumulate dense, settled microbial loads from high-traffic surfaces and remain in prolonged contact with moisture, conditions that can promote fungal proliferation and secondary metabolite production (43, 44). A recent systematic review identified wet mops as a vehicle for microbial redistribution across floor surfaces (43), which, in the context of the dry-sweep-then-wet-mop protocol documented across most sampled schools, likely explains the extreme cytotoxic load in this matrix. Because mops are collected after the application of detergents and disinfectants, however, this load is necessarily both biological and chemical, and the MTT assay does not discriminate between the two contributions; the same reasoning applies to bathrooms (84.6% in both cell lines), the location receiving the most intensive disinfection. Mops should therefore be interpreted as a sentinel of the total toxic burden accumulated at floor level, biogenic and chemical combined, rather than as a specific indicator of microbial contamination. The tight NMDS clustering of EDC and EDCT profiles confirms methodological equivalence between these electrostatic matrices, supporting their interchangeable use in multi-matrix monitoring protocols, while EDCT sampling provides a human-centered complement particularly relevant to characterizing children’s personal inhalation exposure (44).

### 4.5 Fungal strains with reduced antifungal susceptibility and implications for vulnerable school-age populations

Spearman analysis revealed divergent cell-line-specific patterns: fungal growth correlated positively with SK cytotoxicity (SK_5: ρ up to 0.27, *p* < 0.01) but negatively with A549 cytotoxicity at the highest effective concentration (A549_1: ρ as low as −0.22, *p* < 0.05). This opposing directionality likely reflects differential activation thresholds and distinct mechanistic sensitivities of the two cell lines to the complex indoor fungal mixture, consistent with Lu et al.’s observation of non-parallel inflammatory and cytotoxic dose–response relationships across fungal species (40). Two factors may contribute to this inverse pattern. Culture captures only the viable, culturable fraction, whereas cytotoxicity is largely driven by viability-independent material, so low CFU counts and high toxicity may coexist in the same sample; intensive cleaning additionally suppresses culturable growth while adding chemical residue, making cleaning intensity a plausible common driver of both variables. Potential azole resistance showed compound-specific profiles, with voriconazole (VCZ) resistance correlating negatively with A549 cytotoxicity and itraconazole (ITZ) and posaconazole (PSZ) resistance associated predominantly with SK responses. Distinct CYP51A-mediated mechanisms described in the literature (e.g., TR34/L98H vs. TR46/Y121F/T289A) have been linked to different secondary metabolite profiles (15, 16); since resistance was determined phenotypically here and no genotypic data are reported in this manuscript, this interpretation is inferred from published evidence rather than supported by the data presented. Azole-resistant *A. fumigatus* has been detected in environmental reservoirs globally, with hospitals and gardens constituting the main hotspots (14), and recent genomic surveillance in Denmark confirmed overlap between environmental and clinical resistant isolates (45). The detection of reduced azole susceptibility in school dust, and its specific association with cytotoxic activity, adds clinical relevance to the environmental reservoir literature given the potential presence of immunocompromised or asthma-affected children in school settings, for whom exposure to azole-resistant fungal species through settled dust represents potential health impacts (46).

### 4.6 Implications for indoor air quality monitoring and public health

These findings carry three concrete implications for school IAQ policy and monitoring. First, floor mop sampling should be considered as a high-sensitivity, low-cost sentinel approach for monitoring the combined biological and chemical toxic burden at floor level in schools, given its consistently superior capacity to detect cytotoxically active contamination compared to conventional passive matrices. Its integration alongside vacuum filter sampling and EDCT into standardized multi-matrix sampling protocols would substantially improve existing school IAQ surveillance, allowing detection of contamination effects that conventional matrices do not capture. Because settled dust in schools constitutes a mixed biological, chemical and inorganic burden, and because teaching and cleaning staff are exposed to it throughout the working day, dust control in these buildings is equally an occupational health and safety concern (40), and passive multi-matrix sampling offers a practical basis for both surveillance frameworks. Second, the seasonal amplification of A549 cytotoxicity reinforces the need for ventilation standards in schools that account for microbial, not merely CO₂, burden during cold months. The recast EU Energy Performance of Buildings Directive (EU 2024/1275), due for transposition into Portuguese law by 2026, provides a regulatory opportunity to embed these requirements (47). The WHO/Europe IAQ toolbox for children’s settings further recommends scheduling high-emission cleaning outside school hours and using HEPA-filtered vacuum systems (48), recommendations directly supported by the mop cytotoxicity data reported here. Third, the association between potential azole resistance and cell-line-specific cytotoxic activity argues for the inclusion of antifungal susceptibility screening within routine school environmental mycology programmes, a surveillance dimension currently absent from European IAQ regulatory frameworks.

### 4.7 Study limitation

While this study demonstrates significant cytotoxic activity in school dust samples, particularly in bathroom locations and mop matrices, the MTT assay cannot distinguish between cytotoxicity caused by microbial contaminants versus chemical cleaning products. The elevated cytotoxicity observed in bathroom samples (84.6% for both cell lines) and floor mops (94.4% A549; 83.3% SK) may partially reflect the concentrated use of disinfectants and cleaning agents in these environments rather than exclusively fungal contamination. Future studies should incorporate chemical analysis of cleaning product residues alongside microbiological assessments to better characterize the relative contributions of these different contamination sources to the observed cytotoxic effects. One further limitation should be noted: azole resistance was assessed phenotypically in the present work, without molecular confirmation of the underlying mechanisms.

## 5 Conclusion

This study provides the first multi-matrix, multi-season assessment of indoor microbial contamination in Portuguese primary schools using the MTT cytotoxicity assay in two cell lines, fostering assessment of potential microbial contamination effects. Cytotoxic activity was prevalent across all sampling matrices and school locations, with A549 alveolar epithelial cells consistently more responsive than SK renal cells, reinforcing their preferential use as a model for assessing potential respiratory effects in this context. The type of sampling matrix emerged as the primary determinant of cytotoxic variability for both cell lines, with floor mops identified as a sentinel matrix, a finding with direct implications for cleaning protocols and for future monitoring campaigns in schools. Season was a significant secondary modulator exclusively for A549 cells, with cold-season samples exhibiting markedly higher cytotoxic activity, highlighting how reduced ventilation may amplify potential effects from microbial contamination present in settled dust during winter months. Neither geographic context nor school characteristics explained meaningful variation in cytotoxic profiles, indicating that building-level operational factors, cleaning practices and ventilation, are the primary drivers of indoor cytotoxicity observed. The contrasting correlation patterns between fungal contamination, azole resistance markers, and cytotoxic responses in A549 and SK cells underscore the differential sensitivity of the two cell lines to distinct components of the indoor fungal contamination and point to compound-specific toxigenic profiles associated with different antifungal resistance mechanisms. The detection of reduced azole susceptibility in school environments, and its association with cytotoxic activity in cell lines, represents potential effects from exposure to microbial contamination present in settled dust that warrant surveillance in school environments. Collectively, these findings support the integration of passive multi-matrix sampling, including floor mops as a priority matrix, combined with dual cell-line cytotoxicity assessment into standardised school IAQ monitoring protocols. Regulatory frameworks governing children’s indoor environments should incorporate assessment of potential effects from the microbial contamination present in settled dust, using cytotoxicity-based endpoints along with fungal growth, reduced azole susceptibility detection and conventional chemical pollutant thresholds for this vulnerable population.

## Data Availability

The original contributions presented in the study are included in the article/Supplementary material, further inquiries can be directed to the corresponding author.

## Footnotes

1
https://inchildhealth.eu/
