# Supplementary material for: Cytotoxic activity of settled dust in primary schools: a multi-matrix, multi-season screening approach for children’s environmental health
Source: Front Public Health. 2026 Oct 7;14:1946465. doi: 10.3389/fpubh.2026.1946465 (PMC13683694; doi:10.3389/fpubh.2026.1946465)
Supplement: Supplementary file 1 [file Data_Sheet_1.docx]

**Cytotoxic activity of settled dust in primary schools: a multi-matrix, multi-season screening approach for children's environmental health**

**Supplementary materials**

Section S1 — Detailed sampling methodology:

S1.1 School contextual information. Contextual and operational information for each school was collected through a harmonised walkthrough survey, covering cleaning procedures, classroom occupancy, number of staff (teachers and support staff), ventilation type, presence of animals or plants, and average time children spent indoors; full details are reported in Cervantes et al. (2026). In summary, most schools relied primarily on natural ventilation through windows and doors, with only a few employing mechanical ventilation systems. Daily cleaning was performed after school hours by in-house staff in 10 of the 11 schools, with one school outsourcing the service. Floors were generally dry-swept and then wet-mopped, and surfaces such as desks and shelves wiped with cloths and disinfectants; cleaning frequency and product preparation were not consistently reported.

S1.2 Sampling strategy. Environmental sampling procedures, including locations, sample types, and handling protocols, have been previously described in detail in Cervantes et al. (2026). In brief, sampling was conducted in six locations within and around each school — classrooms, canteens, libraries, bathrooms, gymnasiums, and playgrounds — during regular school hours (6–8 h/day), while classes were in session. The passive sampling strategy included four dust-accumulating matrices: vacuum settled-dust filters, electrostatic dust cloths (EDC), electrostatic cloths worn on children's T-shirts (EDCT), and mops used during routine school cleaning. EDCs were left in place for 30 consecutive days to allow long-term dust accumulation. All collected samples were stored in sterile bags and refrigerated (0–4 °C) until further analysis, following validated protocols adapted from Viegas, Gomes, et al. (2022). Additional sampling specifications are presented in Supplementary Table S1.

Supplementary Figure S1 — Study design encompassing indoor environment sampling, dust collection methods, and laboratory analysis for fungal assessment and cytotoxicity evaluation.

Supplementary Table S1: Sampling strategy

| **School** | **Location** | **Filters** | **EDC's** | **EDC-T's** | **Mops** | **TOTAL** |
| --- | --- | --- | --- | --- | --- | --- |
| S1 | Classroom | 2 | 2 | 2 | 2 | 16 |
|  | Canteen | 2 | 2 |  |  |  |
|  | Bathroom | 2 |  |  | 2 |  |
| S2 | Classroom | 7 | 7 | 5 | 2 | 37 |
|  | Canteen | 2 | 2 | 2 |  |  |
|  | Library | 2 | 2 |  |  |  |
|  | Bathroom | 2 |  | 2 | 2 |  |
| S3 | Classroom | 6 | 6 | 6 | 1 | 27 |
|  | Canteen | 2 |  |  |  |  |
|  | Library | 2 | 1 |  |  |  |
|  | Gymnasium | 2 |  |  |  |  |
|  | Bathroom |  |  |  | 1 |  |
| S4 | Classroom | 4 | 4 | 4 |  | 12 |
| S5 | Classroom | 4 | 4 | 2 | 1 | 21 |
|  | Canteen | 2 | 2 |  |  |  |
|  | Library | 1 | 1 |  |  |  |
|  | Gymnasium |  | 1 |  |  |  |
|  | Bathroom |  | 2 |  | 1 |  |
| S6 | Classroom | 4 | 4 | 4 | 1 | 20 |
|  | Canteen | 2 | 2 |  |  |  |
|  | Library | 2 |  |  |  |  |
|  | Bathroom |  |  |  | 1 |  |
| S7 | Classroom | 4 | 3 | 4 | 1 | 21 |
|  | Canteen | 2 | 1 |  |  |  |
|  | Library | 2 |  |  |  |  |
|  | Gymnasium | 2 | 1 |  | 1 |  |
| S8 | Classroom | 6 | 4 | 4 |  | 21 |
|  | Canteen | 2 |  |  |  |  |
|  | Library | 2 | 1 |  |  |  |
|  | Gymnasium | 2 |  |  |  |  |
| S9 | Classroom | 4 | 4 | 4 |  | 17 |
|  | Canteen | 2 |  |  |  |  |
|  | Library | 1 |  |  |  |  |
|  | Gymnasium | 2 |  |  |  |  |
| S10 | Classroom | 4 | 5 | 3 |  | 19 |
|  | Canteen | 2 |  |  |  |  |
|  | Library | 2 | 1 |  |  |  |
|  | Gymnasium | 2 |  |  |  |  |
| S11 | Classroom | 4 |  | 4 | 1 | 15 |
|  | Canteen | 2 |  |  |  |  |
|  | Library | 2 | 1 |  |  |  |
|  | Gymnasium |  |  |  |  |  |
|  | Bathroom |  |  |  | 1 |  |

Supplementary Table S2: PERMANOVA global model for cytotoxicity in A549 cells (dilutions 1–5), including combined factors Context, Matrices, School, Season and Location.

|  | df | Sum of Squares | R^2^ | F | p |
| --- | --- | --- | --- | --- | --- |
| Global Model | 19 | 183568 | 0.365 | 5.652 | < 0.001 * |
| Residual | 187 | 319682 | 0.636 |  |  |
| Total | 206 | 503249 | 1.001 |  |  |
| *Significant differences at the 5% significance level. | | | | | |

Supplementary Table S3: Marginal PERMANOVA analysis to assess marginal effects of factors on cytotoxicity in A549 cells at different dilutions (1 to 5).

| Factor | Df | Sum of Squares | R^2^ | F | p |
| --- | --- | --- | --- | --- | --- |
| Context | 1 | 1417 | 0.003 | 0.8289 | 0.362 |
| Matrices | 3 | 99965 | 0.199 | 19.4918 | <0.001* |
| School | 10 | 20612 | 0.041 | 1.2057 | 0.242 |
| Season | 1 | 35719 | 0.071 | 20.8938 | <0.001* |
| Location | 4 | 6310 | 0.013 | 0.9228 | 0.490 |
| Residual | 187 | 319682 | 0.635 |  |  |
| Total | 206 | 503249 | 1.000 |  |  |
| *Significant differences at the 5% significance level. | | | | | |

SIMPER analysis indicated that differences in cytotoxicity in different dilutions between matrices were mainly driven by dilution 2 (A549_2) and dilution 1 (A549_1), that consistently accounted for the largest proportion of dissimilarity across most pairwise comparisons, together explaining more than 60–90% of cumulative differences depending on the matrices pair. Regarding season, SIMPER analysis revealed that seasonal differences in cytotoxicity in different dilutions (Warm vs Cold) were mainly driven by dilution 1 (A549_1) and dilution 2 (A549_2), which together accounted for approximately 71% of the cumulative dissimilarity between seasons (Supplementary Table S4). These patterns are consistent with PERMANOVA and NMDS results, confirming matrices as the main driver of cytotoxicity variability.

Supplementary Table S4: Cumulative contributions of most influential dilutions in A549 cells

|  | Pair | Cumulative contributions | | |
| --- | --- | --- | --- | --- |
| Matrices | MOP - Filter | A549_2 | A549_1 | A549_3 |
|  |  | 0.352 | 0.625 | 0.889 |
|  | MOP - EDCT | A549_2 | A549_3 | A549_1 |
|  |  | 0.382 | 0.664 | 0.921 |
|  | MOP - EDC | A549_2 | A549_1 | A549_3 |
|  |  | 0.354 | 0.676 | 0.923 |
|  | Filter - EDCT | A549_1 | A549_2 |  |
|  |  | 0.428 | 0.742 |  |
|  | Filter - EDC | A549_1 | A549_2 |  |
|  |  | 0.453 | 0.727 |  |
|  | EDCT - EDC | A549_1 | A549_2 |  |
|  |  | 0.632 | 0.891 |  |
| Season | Warm - Cold | A549_1 | A549_2 |  |
|  |  | 0.403 | 0.707 |  |

Supplementary Table S5: PERMANOVA global model for cytotoxicity in SK cells (dilutions 1–5), including combined factors Context, Matrices, School, Season and Location.

|  | df | Sum of Squares | R^2^ | F | p |
| --- | --- | --- | --- | --- | --- |
| Global Model | 19 | 101384 | 0.294 | 4.091 | < 0.001 * |
| Residual | 187 | 243942 | 0.706 |  |  |
| Total | 206 | 345326 | 1.000 |  |  |
| *Significant differences at the 5% significance level. | | | | | |

Supplementary Table S6: Marginal PERMANOVA analysis for cytotoxicity in SK cells (dilutions 1–5), showing individual effects of each factor.

|  | df | Sum of Squares | R^2^ | F | p |
| --- | --- | --- | --- | --- | --- |
| Context | 1 | 2239 | 0.006 | 1.717 | 0.123 |
| Matrix | 3 | 27861 | 0.081 | 7.119 | <0.001* |
| School | 10 | 21758 | 0.063 | 1.668 | 0.074 |
| Season | 1 | 780 | 0.002 | 0.598 | 0.517 |
| Location | 4 | 9257 | 0.027 | 1.774 | 0.099 |
| Residual | 187 | 243942 | 0.706 |  |  |
| Total | 206 | 345326 | 1.000 |  |  |
| *Significant differences at the 5% significance level. | | | | | |

Considering the only factor with a significant impact on cytotoxicity in SK cells at different dilutions, matrices, the homogeneity of dispersion was not confirmed (p<0.001), indicating that matrix-related differences reflected both shifts in centroid position and differences in within-group variability, particularly for MOP samples. NMDS ordination confirmed these patterns, showing greater dispersion for MOP and more homogeneous profiles for EDC and EDCT (Figure 3b). SIMPER analysis indicated that differences between matrices were primarily driven by dilution 1 (SK_1), with additional contributions from dilution 2 (SK_2) and dilution 3 (SK_3) (Supplementary Table 7).

Supplementary Table S7: Cumulative contributions of most influential dilutions in SK cells

|  | Pairs | Cumulative contributions | | |
| --- | --- | --- | --- | --- |
| Matrices | MOP - Filter | SK_1 | SK_2 |  |
|  |  | 0.516 | 0.783 |  |
|  | MOP - EDCT | SK_1 | SK_2 |  |
|  |  | 0.544 | 0.809 |  |
|  | MOP - EDC | SK_1 | SK_2 |  |
|  |  | 0.445 | 0.706 |  |
|  | Filter - EDCT | SK_1 |  |  |
|  |  | 0.713 |  |  |
|  | Filter - EDC | SK_1 | SK_2 | SK_3 |
|  |  | 0.453 | 0.632 | 0.769 |
|  | EDCT - EDC | SK_1 | SK_2 | SK_3 |
|  |  | 0.472 | 0.642 | 0.770 |

Supplementary Figure S2: Comparison of cytotoxicity at different dilutions between A549 and SK cells, by season.

Supplementary Figure S3: Comparison of cytotoxicity at different dilutions between A549 and SK cells, by context

Supplementary Figure S4: Comparison of cytotoxicity at different dilutions between A549 and SK cells, by location.

Supplementary Figure S5: Comparison of cytotoxicity at different dilutions between A549 and SK cells, by school.
